# Nexus between policy and practice in institutionalization of clinical information systems and knowledge management in developing health systems: Context of tertiary hospitals in Malawi

**DOI:** 10.1371/journal.pdig.0001238

**Published:** 2026-02-17

**Authors:** Edmond C. Kungwalo, Chipo Kanjo, Gregory Kunyenje, Patrick A. Chikumba

**Affiliations:** 1 University of Malawi, Zomba, Malawi; 2 Catholic University, Chiradzulu, Malawi; 3 Malawi University of Business and Applied Sciences, Blantyre, Malawi; The University of Sheffield, UNITED KINGDOM OF GREAT BRITAIN AND NORTHERN IRELAND

## Abstract

In emerging economies, the widespread penetration and mainstreaming of Clinical Information Systems (CIS) and Knowledge Management (KM) practices are challenging and often hindered by fragmented regulatory environments, professional resistance, and cultural misalignment. Drawing on Scott’s institutional theory and its three pillars—regulative, normative, and cultural-cognitive—as an analytical framework, the study aims to inform both policy and practice, contributing to ongoing efforts to strengthen digital health implementation, governance, and institutional capacity in low and middle-income countries (LMIC). The study employed qualitative methods, including analysis of policy documents, key informant interviews, and institutional case study analysis. The findings revealed policy-practice misalignment in the institutionalization of CIS and KM. There is limited engagement of senior management at the facility level in the institutionalization process. Key actors—including government agencies, healthcare providers, regulatory bodies, development partners, and healthcare workers—were identified, along with their roles and responsibilities in shaping institutional outcomes. Application of digital health policies and strategies in Malawi is hindered by limited resources, unclear guidelines, and weak enforcement mechanisms. Consequently, stakeholder engagement is essential for the successful and effective use of technology. These findings suggest that policy–practice gaps are shaped not only by policy design, but also by institutional arrangements and power dynamics within the digital health ecosystem. Thus, successful integration of CIS and KM requires alignment between policy and practice, ongoing training, active participation in system design, and a willingness to integrate digital tools into clinical workflows.

## Author summary

Digital health systems are increasingly promoted as a way to improve healthcare in Malawi, but many of these systems are not fully used once they are introduced. Clinical Information Systems and Knowledge Management tools are often expected to support better decision-making and patient care, yet healthcare workers and facilities face many challenges in using them in daily practice.

In this study, we explored why national digital health policies in Malawi do not always translate into effective use of digital systems at health facility level. We reviewed policy documents and spoke with key people involved in digital health, including policymakers, regulators, development partners, and healthcare workers. We found that limited leadership involvement at health facilities, unclear guidance on how policies should be implemented, and weak enforcement contribute to poor uptake of digital systems. Power relationships and competing priorities among stakeholders also influence how digital health initiatives are carried out. Our findings show that technology alone is not enough to strengthen health systems in Malawi. For digital health investments to succeed, policies must be better aligned with everyday clinical work, healthcare workers need ongoing training and support, and leaders at all levels must actively engage in system design and implementation. These lessons can help improve future digital health planning and data use in Malawi.

## 1 Introduction

Healthcare systems are under increasing pressure to deliver better outcomes despite limited resources, growing populations, and complex disease burdens [1]. One promising approach to improve the quality, efficiency, and effectiveness of healthcare in medical settings is the use of Clinical Information Systems (CIS) and Knowledge Management (KM) tools [2]. Clinical Information Systems (CIS) are a set of interrelated components that work collectively to gather, process, store, and disseminate patient‐related data and information to support clinical decision‐making and direct patient care [3]. While Knowledge Management (KM) is a multifaceted concept with no single definition, but it universally revolves around the creation, storage, use and sharing of information and knowledge.

These technologies support evidence-based decision-making, streamline clinical workflows, and enhance the coordination of care. However, the successful integration of Clinical Information System (CIS) and Knowledge Management (KM) into routine healthcare practices remains limited across much of the Global South [4]. While technical and financial barriers are often cited [5] in application of healthcare practices, institutional factors play an equally critical role in shaping whether these systems are sustainably adopted [6,7]. Efforts to implement CIS and KM are frequently constrained by weak regulatory environments, conflicting stakeholder interests, professional resistance, and cultural misalignment between imported systems and local norms [8]. This gap between policy aspirations and practice outcomes highlights the need for a deeper understanding of the institutional dynamics that govern health system transformation.

This paper draws on [9] three pillars of institutional theory- regulative, normative, and cultural-cognitive- as a lens for analysing how CIS and KM are (or are not) institutionalized in developing health systems. Building on this, [10] propose that when a practice is institutionalized, it presents three traits: *Widespread acceptance* of practice that is perceived as legitimate and necessary. Then s*tructural integration* where practice is embedded into organizational systems and routines. The third trait is h*abitualized behavior*—where practice is performed regularly and almost automatically [10]. These frameworks offer complementary insights. While [9] explains why institutions emerge and persist, [10] offer ways to observe whether institutionalization has truly occurred. This study uses both, to explore the conditions that enable the CIS and KM to take root in real-world healthcare settings. By examining these three pillars, this study aims to do the following:

1. Examine the regulatory, cultural, and value-based factors that promote or hinder the institutionalization of Clinical Information Systems and Knowledge Management in developing economies.
2. Identify key stakeholders and their responsibilities in embedding these systems in healthcare institutions.

Through a qualitative analysis of policy documents, stakeholder interviews, and institutional case study this paper contributes to ongoing conversations about digital health transformation, particularly in low and middle-income countries (LMICs). Ultimately, the goal is to move beyond pilot projects and policy intentions toward lasting, system-wide change.

In the next section, we offer a brief literature review including the concept of institutionalization. This is followed by a conceptual framework guiding the study, which forms the core of our theoretical analysis. We then present our research methodology before moving to a delineation of findings and discussion, and then conclude by highlighting key implications from the research.

## 2 Literature review

The growing interest in digital health tools in low and middle-income countries (LMICs) has led to numerous initiatives aimed at improving data management, service delivery, and clinical outcomes. Among these, Clinical Information Systems (CIS) and Knowledge Management (KM) platforms have shown promise in strengthening decision-making and fostering a culture of evidence-based practice [11,12]. However, their integration into healthcare systems in resource-constrained settings has been inconsistent and often short-lived. Previous studies have tended to focus on technical barriers such as infrastructure, interoperability, and data quality challenges [13,14]. While these factors are important, technological readiness alone is not enough. The sustainability of digital health interventions depends on how well they are aligned with the institutional fabric of the health system [15,16].

Institutions are “the regulative, normative and cultural-cognitive elements of the society that together with associated activities and resources provide stability and meaning to social life” [9, p. 57]. The institutionalization of technology then refers to the extent to which the use of technology becomes *taken for granted* as an ordinary and common part of an organization’s operations [10]. It marks the stage at which technology is so widely used that it becomes the standard method of carrying out tasks [10]. Its influence is now complete within the organization—it is completely embedded in its procedures, and its full influence is felt within the organization [17].

Institutional theory, specifically the work of [9] provides some useful perspectives on this alignment. The model suggests that for any innovation to take root within an organization or system, it must be supported across three domains: formal structures and regulations (regulative), professional roles and social expectations (normative), and the shared understandings or cultural logic of those involved (cultural-cognitive). These pillars have been applied in diverse sectors, including education, governance, and public health [18,19], but their application to the CIS and KM in developing health systems remains relatively underexplored.

Complementing [9], we used [10] three key characteristics that organizations demonstrate when an ethic or practice becomes truly institutionalized: *widespread acceptance, structural integration*, and *habitualized behavior*. Applied to the context of the CIS and KM, widespread acceptance refers to the recognition by healthcare workers and managers of the value and legitimacy of these systems. Without such acceptance, efforts to adopt technology often remain superficial or fragmented. Structural integration means embedding these systems into formal organizational routines, such as clinical workflows, data reporting, and decision-making processes, making them an inseparable part of daily operations. Finally, habitualized behavior signifies that the use of CIS and KM becomes routine and taken-for-granted rather than being viewed as an optional extra task [10,17].

These organizational characteristics help deepen our understanding of institutionalization by focusing on the behaviours and norms that signal genuine integration beyond formal policies or regulations. Health systems in LMICs still grapple with limited acceptance, weak process integration, and inconsistent use of the CIS and KM underscoring the gap between policy ambitions and practical realities [20]. Furthermore, the roles and interactions of key actors—government agencies, healthcare providers, regulators, and development partners are central to how these systems evolve. Literature on stakeholder engagement in digital health highlights the need for trust-building, shared accountability, and long-term commitment, particularly where systems must adapt to local needs and constraints [21,22].

Despite this growing recognition, most existing studies offer fragmented insights, often focusing on one dimension (e.g., policy design or technical deployment) rather than taking a holistic view of institutional dynamics [23,24]. This paper addresses that gap by combining the three-pillar framework with an analysis of stakeholder roles and local context, aiming to generate strategies that are both practically grounded and institutionally sound.

## 3 Conceptual framework

This study draws on [9] institutional theory, which frames institutions as three intertwined pillars: regulative, normative, and cultural-cognitive. These pillars offer a holistic view of social, cultural and policy driving forces that affect the implementation, adoption, and continuous use of CIS and KM in healthcare organizations. The regulatory pillar addresses formal rules, policies, and enforcement patterns, e.g., government regulations, data privacy laws, and national health strategies, which specify the legal framework within which CIS and KM implementation can occur through legal means. The normative pillar covers social norms, professional roles and common expectations that influence organizational behavior and encourage healthcare employees to use new technologies. The cultural‒cognitive pillar presents a mirror view of the shared beliefs and assumptions on the interpretation and appreciation of CIS and KM by the users. Therefore, the regulative pillar is “what should be the case”, the normative pillar is “case that should be there”, and the cultural-cognitive pillar is “what typically happens” [25]. Table 1 shows the pillars, interactions between the pillars and examples from Malawi health system.

**Table 1:** The three pillars of institutionalization (source/author).

Pillar	Function	Interaction with others pillars	Malawi health system example
**Regulative**	Provides formal rules, policies, and sanctions.	Supported by normative acceptance and cognitive legitimacy.	Malawi digital health policy guiding eHealth strategy; Patient data protection and guidelines.
**Normative**	Embodies values and norms of appropriateness.	Reinforced by regulation and internalized through culture.	Adoption of Electronic medical records (EMRs) at Mzuzu central hospital; Standard Operating Procedures (SOPs) for digital data reporting.
**Cultural-Cognitive**	Provides meaning through shared understandings.	Makes rules and norms feel “natural” or obvious.	DHIS2 reporting practices at Zomba and Mzuzu hospitals; routine data review meetings.

**Fig 1:**
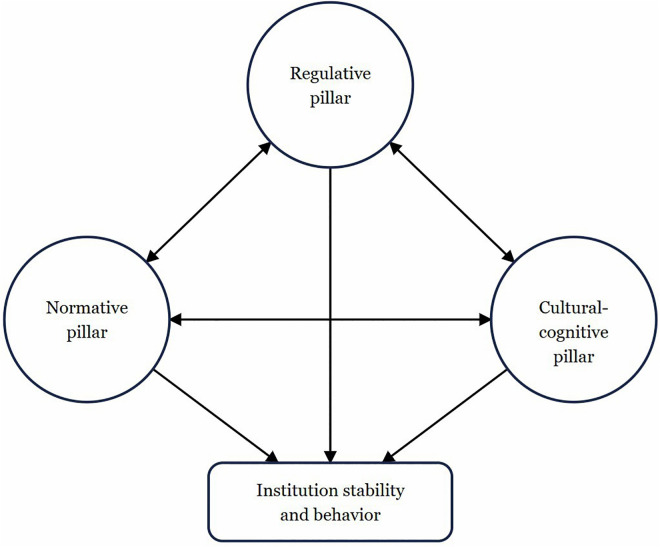
Pillars of institutionalization; adopted from Scott, 2014.

In Fig 1, the pillars support and strengthen one another. They all meet at the centre to support institutional stability and behaviour. Norms and rules influence each other: norms influence beliefs, beliefs influence rule legitimacy and vice versa.

This integrated approach offers a holistic lens to explore how CIS and KM can be effectively embedded in developing health systems, fostering evidence-based practice and long-term health system strengthening. The conceptual framework not only guided data collection and analysis but also helped identify areas for targeted improvements to support CIS and KM institutionalization in Malawi.

To further our comprehension, we include the three organizational characteristics presented by [10] that signal when a practice can truly be considered institutionalized—a psychological contract or widespread acceptance that pertains to the normative and cultural‒cognitive pillars, and measures the degree to which the CIS and KM can be taken as fitting and an asset by doctors and healthcare stakeholders. Organizational commitment or structural integration, which relates to the regulative pillar, emphasizing how the CIS and KM are embedded into formal processes, workflows, and resource allocations within healthcare institutions. Technologically-driven culture or habitualized behavior which indicates that the application of these systems is chalked up and automatic and that the culture has become entrenched at an institutional level beyond casual conformity [10].

This framework integrates the pillars of the structure of institutional factors in [9] with the behaviour features that tend to capture both the external forces of adoption and the internal changes that maintain adoption. The framework also pays attention to major stakeholders, i.e., policymakers, healthcare providers, and funders, whose positions and relationships affect the results of institutionalization [26]. This coordinated mechanism provides a comprehensive perspective for incorporating the CIS and KM in the development of health systems to develop Evidence-based medical practices and strengthen long-term health systems.

## 4 Methodology

A qualitative research design was used in this research to explore key stakeholders and examine factors surrounding the implementation, adoption and continued use of CIS and KM in developing health systems, focusing on Malawi public hospitals as main unit of analysis. The qualitative approach is an appropriate design for studying complex social, cultural, and organizational interactions, which are often ignored by quantitative research methods [27]. A case study approach works well here because it allows for an in-depth look at a contemporary issue within its real-world setting [28].

### 4.1 Ethical consideration

Ethical approval was obtained from the University of Malawi Research and Ethics Committee under protocol P.09/23/297 to ensure it was conducted ethically and in compliance with all state, and local regulations concerning research involving human participants. Before engaging participants, approvals were also obtained from hospital managers. Each participant was requested to sign a concert form before the interview was conducted. To protect the confidentiality of the respondents, their identities were coded. No quotes or statements from the interviews are attributed to specific individuals’ respondent. All interviews (except three) were recorded in audio format for subsequent examination. All digital files, data, transcripts, and summaries were given codes and stores securely and separately from names and other direct identification of participants. Data was on a password-protected computers accessible only to the research team. Participants did not receive any compensation for their participation.

### 4.2 Study context

Health service delivery in Malawi is shaped by a mix of factors - such as limited resources, national policies, and institutional arrangements - so examining these together rather than separately gives a fuller picture of how the system operates. Malawi ranks among the poorest nations in Africa, with an economy that is predominantly dependent on agriculture. In 2021, its GDP per capita was estimated at USD 634.8, the lowest in Southern Africa [29]. The country’s weak economic base significantly constrains healthcare delivery by limiting investment in infrastructure, medical equipment, and essential supplies. Resources that support effective KM - such as reliable computing systems, internet connectivity, and communication technologies - are frequently outdated and inadequate [30]. Delivering medical services under these conditions presents considerable challenges. Hence our decision to use Malawi public health as case study.

Within this case, three public tertiary referral hospitals - Mzuzu central hospital, Kamuzu central hospital, and Zomba central hospital - were examined as embedded units of analysis, consistent with an embedded case study design [28]. Tertiary hospitals serve as referral hubs within the public health system and play a central role in policy implementation, management of complex conditions, and coordination of care across levels. Examining multiple hospitals allows comparison across settings, strengthening analytic depth and credibility while maintaining focus on the national public health system as a single case. This approach also aligns with the view that well-chosen cases can generate insights that extend beyond individual study sites [31].

### 4.3 Data collection

Data were collected from three public tertiary referral hospitals: Zomba central hospital in the southern region, Kamuzu central hospital in the central region, and Mzuzu central hospital in the northern region. Malawi has four public tertiary referral hospitals, with one located in each region, except for the southern region which has two. The hospitals were selected to ensure representation from each administrative region of the country. One tertiary hospital was therefore included from each region. In the southern region, where two tertiary hospitals are available, one hospital was selected based on feasibility and accessibility. This approach reflects a combination of regional representation and convenience. Although the use of convenience sampling may limit the generalisability of the findings, the inclusion of tertiary referral hospitals from all regions provides a reasonable national perspective on care within Malawi’s public tertiary health system.

Data collection took place between February, 2025 and August 2025. A total of 36 interviews were conducted. The interviews were done by the first author. After establishing rapport with one doctor, that respondent assisted by referring the researcher to other willing doctors for formal interviews. Some interviews took place in the doctors’ offices, while others occurred at mutually agreed-upon locations, with each interview lasting approximately 35 min. A semi-structured interview guide was used, covering topics related to use of technology in patient treatment, knowledge management practices, institutional and organisation challenges in application of IT and KM in healthcare and healthcare policy implementation.

#### 4.3.1 Policy document analysis.

We conducted a systematic review of relevant policy documents. The documents analysed included: Malawi digital health policy, Health sector strategic plan III, Data Protection Act, National Health policy, and Digital Health Strategy. Documents were selected based on relevance for example national health policy, hospital management guidelines, or strategies implemented from 2019 to date. Each document was reviewed for content related to the study objectives, and findings were integrated with interview data to provide a comprehensive analysis of policy and practice. Only relevant policy documents, and regulatory frameworks were scrutinized to clarify the formal regulatory environment and policy purposes on which the CIS and KM are based.

#### 4.3.2 Interviews.

In addition to policy documents review, we conducted interviews with doctors and policy makers. While the research is focused on the doctors, it is the hospital managers and ministry of health who have the power in establishing the policies and guidelines, and often the actual tools being used, so interviews with these officers were considered appropriate. The interview was semi-structured, to obtain full information. The interviews were audio-recorded with participants’ consent, transcribed verbatim, and anonymized to maintain confidentiality. The Interview guide remained unchanged throughout the data collection; however, in line with the concept of constant comparison [32] some questions were added to the guide based on insights from the initial interviews. The aim of the semi-structured interviews was to question the normative and cultural factors that influence institutionalization and to help elucidate the roles and responsibilities of stakeholders.

#### 4.3.3 Site visits.

Three publicly-owned tertiary hospitals with varying degrees of CIS and KM usage were analysed to observe institutionalization at an operational level: visits on site, systematic observations, and interviews with representatives of a sphere under analysis, which served as the means to determine the level of acceptance, incorporation, and regular use of these digital tools. Doctors and key stakeholders, including government officials and healthcare managers, were interviewed. Doctors were included as frontline users of CIS and key actors in KM processes within public hospitals, while Ministry of Health officials were selected for their roles in policy development, oversight, and resource allocation.

### 4.4 Sampling

Purposive non-probabilistic sampling technique was employed to select participants. Random sampling was unnecessary since the focus was on doctors’ perspectives rather than statistical significance. Purposeful sampling is a widely used technique in research for the identification and selection of “information-rich” cases for the most effective results. It involves identifying and selecting respondents who are especially knowledgeable or experienced with a phenomenon of interest [33]. This technique was suitable for this study as it was intended to provide an in-depth understanding of the practice of CIS and KM tools.

The sample size was not predetermined but determined based on theoretical saturation [34,35]. The saturation point is the stage in data collection where new data no longer provide novel insights into addressing the research. The authors concluded that the saturation point was reached with 36 respondents, comprising 26 medical doctors, 5 Ministry of Health officials, and 5 hospital managers. It is not unusual for saturation to occur with a relatively small number of interviews, particularly when analysing a single case. A similar occurrence was observed in a study by [36], on Knowledge sharing in the pharmaceutical industry, where saturation was achieved with seven interviews.

The inclusion criteria for doctors were: (i) employment in a public tertiary hospital, (ii) direct involvement in clinical decision-making and/or CIS use, and (iii) at least two years of work experience to ensure adequate familiarity with institutional processes. For Ministry of Health officials, inclusion required: (i) direct involvement in health information systems, digital health, or policy formulation, (ii) work within departments linked to CIS or KM initiatives, and (iii) a position providing insight into national-level policy implementation.

To provide a clear overview of the study participants and the distribution of interviews across hospitals, Table 2 summarizes the number of participants by category (doctors, Ministry of Health officials, and hospital managers) and by hospital. Details of the policy documents reviewed, including the type of document, year of publication, are presented in Table 3.

**Table 2:** Summary of study participants.

Healthcare organisation	Informants	Number of informants	Area of interest
Kamuzu Central Hospital	Medical Doctors	16	• Tools used in patient management, collaboration, and knowledge sharing. • Challenges faced when searching for patient information and current medical knowledge. • Policies, rules, and regulation regarding use of technology and knowledge management in healthcare.
	ICT Officer	1	
	Hospital Director	1	
Mzuzu Central Hospital	Medical Doctors	5	
	ICT Officer	1	
	Hospital Director	1	
Zomba Central Hospital	Medical Doctors	5	
	ICT Officer	1	
Min of Health - Digital Health Department	Director of Digital health	1	
	Deputy Director Malawi health Information Systems	1	
Min of Health M&E Department	Deputy Director	1	
	Senior officer M&E	1	
Medical council of Malawi	Director of Regulatory	1	
Total			36

**Table 3:** Documents reviewed.

Document	Document Type	Purpose	Publication Date
Malawi digital health policy	Policy	It offers a strategic direction for the development, utilisation, and regulation of digital health solutions in Malawi	2024
Health sector strategic plan III	Strategic plan	The strategic plan focuses on achieving high-quality, integrated, and equitable healthcare delivery, with specific emphasis on governance and leadership improvements at all levels of the health sector.	2023
Data Protection Act	Act of Parliament	Protects the personal data and privacy of individuals by regulating how personal information is processed and handled by public and private entities.	2024
National Health policy	Policy	The policy establishes the legal foundation for health service delivery, including the integration of ICT into health systems	2018
Digital Health Strategy	Strategy	Outlines a roadmap for harmonised country-led digital health initiatives that support efficient service delivery at all levels of the health system.	2020

### 4.5 Data analysis

The data were analysed thematically [37], guided by the conceptual framework presented in section 3 . The coding exercise focused on identifying themes related to the regulative, normative, and cultural‒cognitive pillars, as well as Carlson and Perrewe’s institutionalization characteristics (acceptance, integration, and habitual behaviour). Stakeholder roles and interactions were mapped and analysed. The analysis aimed to triangulate findings across data sources to build a nuanced understanding of how institutional factors enable or hinder the embedding of CIS and KM in developing health systems.

To strengthen the credibility of our analysis, two authors EK and GK, first coded a subset of transcripts independently and then met to compare interpretations. When differences arose, the authors discussed openly and returned to the original data to understand why the readings diverged. These conversations continued until a shared interpretation that reflected the participants’ actual words and experiences was reached. Throughout the process, we repeatedly revisited the full dataset and compared emerging ideas across transcripts to ensure that the themes were shaped by the data itself rather than by authors own expectations or theory.

## 5 Results

The study revealed several interconnected concepts through the analysis of interviews and policy documents, shedding light on institutionalisation of CIS and KM in developing health systems especially in the examined health facilities. We now present the findings in relation to the three pillars of institutionalisation.

### 5.1 Regulative pillar: Policy and governance

The analysis revealed that the Ministry of Health (MoH) has established policies and regulatory frameworks aimed at promoting digital health and KM. Within the MoH, two divisions oversee these areas: the research division, responsible for KM, and the digital health division, responsible for digital health initiatives. However, enforcement and implementation of both KM and digital health initiatives often remain inconsistent. Stakeholders noted that although digital health strategies exist, their application is hindered by limited resources, unclear guidelines, and weak enforcement mechanisms. A senior officer at one hospital explained:

*“They [the ministry] talk about digital health strategies at the national level, but here on the ground, it is not easy to see them working. We lack the resources — like internet access, computers, and even training. The guidelines are not well explained to us, so we do not always know how to use these systems properly. And because there is no strong follow-up, we do not implement.”- Doc 06*

This concern came up often in conversations with participants at the facility level. Many spoke about hearing national discussions on digital health but struggling to see how these strategies translated into their daily work. They described practical constraints such as unreliable internet, lack of equipment, and limited training, alongside uncertainty about how to apply policy guidelines in practice. Several participants also pointed to weak follow-up and support, which left them unsure how—or whether—to fully implement digital health systems. These experiences highlight how national strategies, without sustained local support, remained difficult to embed in routine practice.

Our observation in all three facilities visited show lack of equipment. For example, all consultation rooms at both Kamuzu central and Zomba central hospital had no computers, internet access was limited, only available to administration staff. A clear sign the tools necessary for KM were limited.

Examination of digital health policy shows that the policy requires countrywide harmonization of digital systems and comprehensive coverage across all areas of health service delivery. However, the hospitals visited lacked hospital-wide CIS, instead they rely on stand-alone, program-specific applications. Infrastructure gaps further undermined policy implementation, as the facilities did not have stable internet connectivity or adequate hardware, despite policy commitments to provide the foundational resources needed for digital transformation. Moreover, the study revealed that existing systems operated in isolation, contrary to policy provisions on interoperability intended to support continuity of care. Facilities continued to deploy software that failed to meet established interoperability standards, even though such requirements are clearly articulated in national digital healthy policy and strategic plan.

### 5.2 Normative pillar: Professional norms

Normative influences emerged as central to the adoption of CIS and KM. Although the Ministry of Health (MoH) has established a dedicated digital health division to advance e-health initiatives, senior leadership at the facility level remains only marginally involved in the digitalization process. An MoH official explained:

*“The Digital health division is an important division for the ministry, but the real challenge is at the facility level. Senior leaders are not yet fully invested in the process. They may attend meetings or acknowledge the importance of digital health, but in practice, their engagement and commitment remain quite limited. This slows down the pace of adoption.” -MoH 02*

This limited engagement was identified as a major barrier to the effective implementation of e-health systems. Doctors’ acceptance of these systems depended heavily on leadership endorsement and peer support. Resistance also emerged due to concerns about increased workload and insufficient training. Many viewed the systems as added workload, with limited training leaving them unsure how to use the tools. One participant said:

*“Honestly, when they introduce these computer systems, a lot of us are hesitant. It just felt like more work on top of everything else, and we do not really get the training we needed. So yeah, there is some kind of pushback.” - Doc 17*

In contrast, those who received thorough orientation and mentorship adopted the systems more smoothly. As one noted:

*“Once we had proper guidance and saw how the system could actually reduce our reporting time, most of us started using it regularly.” – Doc 09*

These accounts indicate that resistance reflects gaps in implementation and support rather than inherent flaws in the digital health systems themselves. Our analysis highlighted several everyday organisational realities that make it difficult for hospitals to fully embed technology and KM practices. In the three facilities visited, staff described a limited culture of sharing what they know. This is what one doctor said.

“*Experiences are rarely documented. We do not have a system to record strange experiences or lessons learnt, except for morning handover meetings and occasionally journal clubs”- Doc 09*

Sentiment that doctors expressed show that they have few opportunities to openly discuss challenges. When new digital tools are introduced, they often complicate rather than streamlined work, as they are added on top of existing paper systems and create extra steps, duplicated tasks, and bottlenecks in already busy workflows. We also found that hospitals lack clear structures for tracking how new technologies or KM efforts were being implemented. Without defined indicators, feedback processes, or accountability mechanisms, teams struggled to identify what was working, what needed improvement, and how to adjust their practices over time.

### 5.3 Cultural-cognitive pillar: Shared beliefs and values

The study found that the extent to which CIS and KM were internalized varied considerably across hospitals. In some settings, such as Mzuzu hospital, the Electronic Medical Record (EMR) system was embraced as a tool for improving care quality. In other facilities, skepticism persisted, often linked to a lack of perceived relevance or misalignment with clinical realities.

At Mzuzu hospital, we found that if a patient’s diagnosis and prescribed medication were not recorded in the EMR, the patient could not receive medicine from the pharmacy, as the drug dispensing system is directly linked to the EMR system. Drugs are only dispensed after scanning the barcode on the patient’s health passport, which is updated through the EMR system. This setup encourages doctors to record patient visits, diagnoses, and prescriptions directly in the system. However, some doctors continue to record patient visits manually in the health passports, either due to resistance to using the CIS, lack of computers or indeed lack of computing skills. When visits are recorded manually, patients must rely on data entry clerks to transfer the information from the health passports into the electronic system, a process that is prone to errors.

Initially, CIS was understood primarily as digital tools for data storage and processing. Over time, their broader strategic value became more apparent. CIS is now increasingly recognized not only as technological tools, but also as integral resources capable of enhancing operational efficiency and improving patient outcomes. A doctor at Mzuzu hospital explained:

*“We used to see the EMR system as just one of these other software, but now it is really part of how we run things. It helps streamline operations and definitely helps us provide better patient treatment.” – Doc 18*

This sentiment was commonly expressed by doctors at Mzuzu, though it was not shared by all participants. These observations indicate that acceptance and utilization of CIS and KM is influenced by how staff perceive their practical value and contribution to daily workflows and overall facility performance.

### 5.4 Institutionalization characteristics: Acceptance, Integration, and Habit

Although all three hospitals operated under the same national digital health policy and framework, their experiences on the ground differed markedly. Our analysis revealed clear variation in the stages of institutionalisation across the three facilities. At Mzuzu hospital, EMR use had progressed to habitualized stage, where engagement with the system had become a routine and taken-for-granted part of daily clinical and administrative work. Doctors and Management Information Systems (MIS) staff routinely entered patient data, periodic data quality checks were conducted, and reports generated from the system were discussed and used to inform local decision-making.

*“Here, you cannot really work without the system. Every patient is in the EMR, and we use the reports when we are reviewing our performance. It has become part of how we work.” - Doc 21*

This pattern of use, reflects a deeper embedding of the EMR within everyday workflows and suggests a more mature level of institutionalisation. Several respondents attributed Mzuzu hospital’s relative success to a combination of sustained senior management support, donor-funded investments in infrastructure, and the presence of dedicated IT personnel who were able to address technical challenges as they arose.

*“Management was very clear from the beginning that the system [EMR] was not optional. They supported training and made sure there was someone on site to fix problems.” - HM 02*

A further distinguishing factor was workflow integration. At Mzuzu, the drug dispensing system is integrated with the EMR, such that medication could not be issued unless a patient’s diagnosis had first been captured in the system. This integration effectively made EMR use unavoidable, reinforcing routine engagement and supporting higher levels of user acceptance.

*You cannot dispense drugs unless the diagnosis is entered in the system. Everyone must use the system, whether they like it or not.”* – *HM 02*

In contrast, Kamuzu and Zomba Central hospitals exhibited much weaker levels of institutionalisation. In both facilities, CIS implementation remained limited to isolated pilot initiatives or confined to specific functions, most notably patient registration. EMR use in these settings was often described as something undertaken primarily to meet external reporting requirements rather than as a tool that supported clinical or managerial work.

*“We only use the system when registering patients. After that, everything else is still done on paper.” - IT 03*

Similarly, staff at Kamuzu Central hospital emphasised that CIS use was primarily driven by external reporting requirements rather than local needs:

*“Other than the EMR which is used for patient registration, we have DHIS 2. The system is mainly for reporting to headquarters. We don’t really use the information ourselves.” - IT 02*

Data entry was irregular, system outputs were rarely discussed locally, and staff reported difficulties in translating available information into actionable knowledge. This localised and narrow use reflects an early or partial stage of institutionalisation, where the technology had not yet been assimilated into broader organisational processes. Table 4 provide comparative summary of CIS and KM institutionalisation in the three hospitals. This comparison is across five dimension- EMR implementation status, system integration, KM practises, leadership support, and technical capacity.

**Table 4:** Comparative overview of CIS and KM Institutionalisation across the three hospitals.

Dimension	Mzuzu Central	Kamuzu Central	Zomba Central
**EMR implementation status.**	Fully operational in key departments.	Fragmented, limited use, largely paper-based.	Fragmented, limited use, largely paper-based.
**System integration**	Moderate (clinical, pharmacy and reporting modules linked).	Very low.	Very low.
**KM practices**	Routine data review meetings, local reporting, and journal clubs.	Minimal formal KM activities. Ward rounds and journal clubs.	Minimal formal KM activities. Ward rounds.
**Leadership support**	Strong senior management backing.	Weak.	Mixed.
**Technical capacity**	Dedicated IT staff	Dedicated IT staff	No dedicated IT staff.

### 5.5 Stakeholder roles and interactions

The study observed significant collaboration and donor support in the development and institutionalization of digital health. During a digital health Technical Working Group (TWG) meeting, several local and international organizations were present, highlighting the complexity of stakeholder roles and interactions involved in the institutionalization of technology in healthcare. As digital health tools have become more widely adopted, the number of actors engaged in their design, financing, and implementation has grown substantially.

While this expansion reflects strong interest and investment in digital health, it has also exposed weaknesses in governance and coordination. In practice, institutions often move forward independently, leading to fragmented implementation, overlapping initiatives, and repeated efforts to address similar challenges. Without clear governance arrangements to align roles, responsibilities, and mandates, these parallel activities work at cross-purposes, limiting coherence across the health system and reducing the overall impact of digital health interventions.

Within this complex landscape, major stakeholders included the Ministry of Health (MoH), the Medical Council of Malawi, health facility management, health workers, and development partners. The MoH plays a central role in policy development, strategic planning, and resource allocation. By integrating CIS and KM into national digital health policies, the Ministry sets direction and promotes alignment across all levels of the health system. The MoH has developed several documents guiding and directing digital health initiatives in the country.

Complementing this role, the Medical Council of Malawi supports institutionalization by incorporating CIS and KM competencies into professional standards and licensing requirements, thereby influencing long-term behavioural and cultural change among medical doctors. At the operational level, hospital managers are critical for implementing CIS and KM, allocating resources, leading change management processes, and adapting systems to their facilities’ specific needs. Doctors, as primary users, are essential for successful adoption. Their engagement through training, participation in system design and feedback, and integration of digital tools into clinical workflows is pivotal for sustaining these systems. Development partners contribute technical expertise, funding, capacity-building support, infrastructure development, and support scaling-up of successful pilots at national level. Overall, stakeholder engagement emerged as a critical enabler, with collaboration among government bodies, healthcare providers, users, and funders shaping institutional outcomes.

## 6 Discussions

The findings highlight a persistent gap between policy formulation and the practical implementation of digital health and KM initiatives. Similar gaps have been documented in previous studies [38,39] which note that without sufficient operational capacity, policies and regulations rarely translate into effective institutional change [40]. This aligns with Scott’s regulative pillar [9] which emphasizes that formal rules require enforcement and supportive structures to guide behaviour, as well as Carlson and Perrewe’s [10] concept of structural integration, which stresses the need for policies to be embedded within routine organizational processes.

While this policy–practice misalignment echoes existing literature, our study provides insight into the specific mechanisms through which this gap emerges in the Malawian context. Interviews and observations suggest that although national digital health policies articulate clear strategic priorities, their translation into practice is constrained by limited institutional coordination, overlapping mandates among implementing actors, and weak enforcement of governance arrangements. Development partners and implementing organizations often pursue project-specific priorities that are only loosely aligned with national strategies, contributing to fragmented implementation at the facility level. Unlike contexts where strong regulatory oversight moderates these dynamics, Malawi’s rapid digital health expansion has occurred in the absence of commensurate governance capacity, exacerbating divergence between policy intent and operational realities. These findings suggest that policy–practice gaps are shaped not only by policy design, but by institutional arrangements and power dynamics within the digital health ecosystem.

At the facility level, the internalization of CIS and KM varied considerably across hospitals, reflecting differences in staff perceptions, leadership support, and engagement with these systems. In hospitals such as Mzuzu, where EMR systems were more fully embraced, staff reported tangible benefits related to care quality and workflow efficiency. In contrast, skepticism in other facilities appeared to stem not only from system limitations, but from perceptions that CIS and KM initiatives were poorly aligned with everyday clinical workflows. In the Malawian context, this divergence appears to be influenced by differences in training exposure, leadership commitment, and the extent to which frontline staff were involved in system implementation. Facilities where EMR use was embedded into routine clinical processes reported greater acceptance, suggesting that technology adoption is shaped as much by organizational integration as by system availability.

This finding extends existing literature by demonstrating that resistance to CIS and KM in low-resource settings cannot be attributed solely to infrastructure or capacity constraints. Rather, our study highlights the importance of perceived strategic relevance, particularly the extent to which systems support clinical decision-making and workflow efficiency. These insights suggest that improving CIS and KM uptake in Malawi requires presenting and implementing these systems as integral components of clinical practice, rather than as parallel or externally driven data collection tools [41]

Significant variation was also observed in how hospitals perceived and internalized CIS and KM. In facilities such as Mzuzu, where these systems were viewed as contributing to improvements in care quality, institutionalization appeared more advanced. By contrast, skepticism at Zomba and Kamuzu Central Hospitals was often linked to limited awareness of system value or poor contextual alignment. This variation mirrors findings by [42,43] who emphasize that perceptions of relevance and clinical fit play a central role in shaping technology uptake. The gradual shift from viewing CIS and KM primarily as data tools toward recognizing them as strategic instruments underscores the importance of cultivating shared meaning around digital systems, consistent with Scott’s cultural–cognitive pillar [9].

The findings further illustrate that hospitals occupy different positions along the institutionalization continuum described by Carlson and Perrewe [10]. Facilities where CIS use has become habitualized demonstrate stronger structural integration, reflecting both organizational commitment and the normalization of digital practices among users. In contrast, facilities with partial or departmental adoption reveal incomplete institutionalization, often characterized by fragmented implementation and limited engagement. The reciprocal relationship between user acceptance and institutional depth is evident: as CIS becomes embedded in daily practice, acceptance strengthens, which in turn reinforces sustained use and deeper organizational assimilation.

This highlights the importance of fostering both structural and behavioural integration to support long-term digital transformation. This finding reflects the processes described by [44] who emphasize how shared professional norms shape the acceptance and routinization of new technologies. These dynamics illustrate Carlson and Perrewe’s [9] idea of widespread acceptance, where institutionalization hinges on shared professional values and the perceived legitimacy of new technologies.

At the same time, hospital environments remain insufficiently equipped to support effective CIS and KM adoption. A limited culture of knowledge sharing constrains collective learning, while poorly integrated digital tools can disrupt workflows rather than support them. The absence of clear monitoring and evaluation mechanisms further limits hospitals’ ability to identify implementation challenges and make timely adjustments, undermining sustainability. Together, these findings suggest that strengthening organizational foundations—through supportive leadership, workflow alignment, and effective oversight—is critical for embedding CIS and KM into routine hospital practice.

Finally, the findings indicate that institutionalizing CIS and KM extends beyond introducing new technologies or developing supportive policies. While Ministry of Health officials emphasized policy frameworks and strategic priorities, doctors and hospital managers highlighted practical challenges, including infrastructure limitations, insufficient training, and disruptions caused by poorly integrated systems. This divergence reinforces the misalignment between policy ambitions and frontline realities, underscoring that successful institutionalization is a complex, multi-layered process. Coordinated efforts across stakeholders—combining policy direction, operational support, user engagement, and sustained donor alignment—are essential to ensure that CIS and KM are meaningfully embedded in everyday clinical practice and sustained over time.

## 7 Conclusion

Institutionalizing CIS and KM in developing countries is a complex, process. It requires more than technology or policy— it demands alignment between systems, values, and the daily realities of healthcare workers. This study has shown that successful institutionalization depends on the presence of regulatory support, professional acceptance, and cultural fit. By using Scott’s institutional pillars and Carlson and Perrewe’s characteristics of institutionalization as guiding frameworks, we can better understand where the CIS and KM stand within healthcare systems and how to strengthen their foothold. When supported by meaningful policies, inclusive leadership, and stakeholder collaboration, these tools have the potential to not only support evidence-based medicine but also become part of the foundation of healthcare delivery itself.

These findings affirm that effective institutionalization of the CIS and KM requires integrated efforts. Taken together, these findings highlight that successful institutionalisation of CIS is not determined solely by the availability of technology or national policy mandates. Rather, it is shaped by local organisational conditions, including leadership support, workflow integration, technical capacity, and the extent to which systems are embedded in everyday practices that make their use meaningful to staff.

## 8 Recommendations

The institutionalization of clinical information systems (CIS) and knowledge management (KM) in Malawi faces several challenges, including inconsistent policy enforcement, fragmented systems, limited infrastructure, variable adoption, and low engagement from facility leadership. To address these issues, the Ministry of Health (MoH) should provide clear operational guidance, strengthen coordination between divisions, enforce interoperability standards, and ensure foundational infrastructure is in place. Leadership responsibilities for CIS and KM should be explicitly incorporated into performance metrics, while hospital administrators should align local practices with national policies, assign clear responsibilities for digital initiatives, prepare workflows and resources, and support early adopters in mentoring peers to build buy-in across staff.

Development partners should focus on supporting MoH-led implementation rather than creating parallel systems, co-invest in essential infrastructure alongside digital tools, and facilitate peer learning and cross-facility exchanges to share successful practices. Taken together, these steps—policy reinforcement, leadership engagement, context-sensitive adaptation, and targeted support—can help translate national strategies into practical actions that improve continuity of care, strengthen service quality, and ensure the sustainability of digital health initiatives.

### 8.1 Limitations

This study offers an in-depth look at the factors shaping the adoption and continued use of CIS and KM in developing health systems, but there are some important limitations. The qualitative design and purposive sampling mean the findings may not be fully generalizable, and those who chose to participate may have been more positively inclined toward digital systems. Relying on self-reported data also introduces the possibility of social desirability or recall bias, and our presence during site visits may have influenced staff behavior, though we triangulated observations with interviews and documents to reduce this effect. Some interviews were conducted in both English and local language (Chichewa), which may have limited subtle expressions, and data collection took place post-COVID, when workflows and priorities may have been shaped by the pandemic. Finally, as researchers with prior engagement in health facilities, our perspectives may have influenced interpretation; we reflected on this through notes, peer discussions, and multiple coders. Despite these limitations, combining document analysis, interviews, and case studies provides a rich and credible understanding of institutionalization processes.
